# Serum pentraxin 3 as a biomarker for prognosis of acute minor stroke due to large artery atherosclerosis

**DOI:** 10.1002/brb3.1956

**Published:** 2020-11-18

**Authors:** Yan Zhang, Haijie Hu, Chong Liu, Juan Wu, Shanshan Zhou, Tingting Zhao

**Affiliations:** ^1^ Department of Neurology First Affiliated Hospital of Harbin Medical University Harbin China

**Keywords:** C‐reactive protein, minor stroke, pentraxin 3, prognosis

## Abstract

**Objectives:**

Pentraxin 3 (PTX3) may reflect local inflammatory status in tissues and thus serve as a potential biomarker of inflammation. Here, we investigated the utility of serum PTX3 as an index for assessing the 90‐day prognosis of acute minor stroke patients.

**Materials & Methods:**

Acute minor stroke patients (*N* = 241) and matched healthy control subjects (*N* = 241) were prospectively recruited. Clinical, laboratory, and imaging data were assessed. Blood samples were collected within 48h after acute minor stroke onset and serum PTX3 levels were determined.

**Results:**

Significant increases in stroke patients versus controls were obtained for serum PTX3 (3.14 ± 1.23 vs. 2.44 ± 0.74 ng/ml; *p* < .001) and C‐reactive protein (CRP – 1.53 ± 0.38 vs. 1.35 ± 0.35 μg/ml; *p* < .05). Among the four stroke subtypes, as defined by modified Trial of Org 10172 in Acute Stroke Treatment classification, there were no statistically significant differences in serum PTX3 levels (*p* > .05). Multivariate logistic regression analysis revealed that serum PTX3 and LDL cholesterol could predict unfavorable outcomes at day 90 in Large Artery Atherosclerosis (LAA) patients.

**Conclusions:**

Serum Pentraxin 3 may serve as an independent predictor for an unfavorable outcome in the LAA subtype of acute minor stroke and may possess a superior prognostic value as compared to CRP in this LAA subgroup.

## Graphical abstract

Serum pentraxin 3 (PTX3) levels were significantly increased in minor stroke patients. No significant differences in serum PTX3 levels were obtained among stroke subtypes. Serum PTX3 may serve as a predictor for an unfavorable outcome in the Large Artery Atherosclerosis subtype.

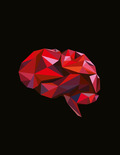

## 1 INTRODUCTION

Pentraxin 3 (PTX3), an identified member of the pentraxin superfamily, includes C‐reactive protein (CRP) and serum amyloid P. In contrast to CRP, which is produced by the liver, PTX3 is produced in response to inflammatory stimuli within a variety of cell types and tissues, in particular within the vasculature (Garlanda et al., 2005). PTX3 may reflect local inflammatory status in tissues and may thus serve as a biomarker of inflammation (Casula et al., 2017). Results from animal studies have indicated a beneficial role of PTX3 in atherosclerotic plaque development and vulnerability (Shiraki et al., 2016). PTX3 also promotes sustained long‐term recovery of cerebral blood fluid, angiogenesis, and neuronal viability after cerebral ischemia. As a key regulator of angiogenesis, PTX3 is emerging as a promising target for cerebrovascular repair after stroke (Rajkovic et al., 2018). However, results from clinical studies have so far provided contrasting results, with these findings indicating a debatable role of PTX3 as related to active mediation of endothelial dysfunction, atherosclerotic plaque vulnerability, and worsened outcomes after ischemic events (Katakami et al., 2013).

Minor strokes represent the most common manifestations of acute cerebrovascular disease, accounting for approximately 50% of all ischemic strokes (Weitzel‐Mudersbach et al., 2013). The definition of “minor stroke” as employed by the National Institute of Health Stroke Scale (NIHSS) involves scores ranging from 0 to 3, as used by Fischer et al. (2010). Patients with acute minor ischemic stroke are at a high risk of recurrent stroke and cardiovascular events (Khanevski et al., 2018). Inflammation plays an important role in the development of atherosclerosis, and inflammatory mediators can synergistically amplify traditional risk factors of acute ischemic stroke. In fact, increased inflammatory activity is associated with the progression and the future risk of acute ischemic stroke (Libby, 2012). However, the role of inflammatory mediators in acute ischemic stroke is less well understood and routine measurements of inflammatory markers are not supported by international guidelines (Li et al., 2019).

Only a few original articles related to PTX3 and acute ischemic stroke exist in the literature and the relationship between PTX3 and clinical outcomes in acute minor stroke remain unknown. The goals of this study were to evaluate the potential association between high levels of serum PTX3 and an unfavorable prognosis of acute minor stroke as well as to investigate the clinical significance of PTX3 as related to acute minor stroke. To address these issues, we assessed the utility of serum PTX3 levels for assessment of the 90‐day prognosis for acute minor stroke patients.

## 2 MATERIALS & METHODS

### 2.1 Data availability

The authors will allow the data, detailed methods, and all other study materials to be available to any investigators interested in reproducing the analysis in this report.

### 2.2 Study design and patients

The study protocol was approved by the leading Ethic Committee of the First Affiliated Hospital of Harbin Medical University (reference number#201894) and was performed in accordance with the Declaration of Helsinki and its subsequent amendments. Records of acute minor stroke patients admitted consecutively to the Department of Neurology of the First Affiliated Hospital of Harbin Medical University over the period from January to May in 2018 were prospectively reviewed. Written informed consent was obtained from each patient before entry into the study. Stroke was defined as the sudden onset of a focal neurological deficit lasting for more than 24 hr. The diagnosis of cerebral infarction was achieved with use of neuroimaging including CT and MRI. For the purpose of this analysis, we included patients aged ≥18 years with: (a) clinical diagnosis of ischemic stroke and (b) National Institute of Health Stroke Scale (NIHSS) Brott et al., 1989 baseline 0–3. Exclusion criteria were as follows: (a) History of acute coronary syndrome within 3 months preceding enrollment, (b) Chronic or systemic inflammatory diseases, (c) Peripheral artery disease, (d) Diseases such as heart, renal or hepatic failure; malignancy or collagen disease, (e) Infections, (f) Use of anti‐inflammatory medication within the past 2 weeks and (7) Drug addiction/abuse.

Subjects receiving general physical examinations at the medical center of the First Affiliated Hospital of Harbin Medical University were enrolled as the control group. The subjects recruited as controls showed no history of cardiovascular diseases (e.g., stroke, coronary heart diseases, or atrial fibrillation) and were age‐ and gender‐matched with the acute minor stroke patients. The above‐described exclusion criteria as described for the patients with acute minor stroke were also applied in the selection of the controls.

All ischemic stroke patients were further sub‐classified according to a modified Trial of Org 10172 in Acute Stroke Treatment (TOAST) criteria Adams et al., 1993. Specifically, we combined “Stroke of other determined etiology” and “stroke of undetermined etiology” into the single category of “other types of brain infarction” (OT). Therefore, the final categories then included in our study were as follows: large artery atherosclerosis (LAA), small‐artery occlusion (SAO), cardioembolism (CE), and OT (including determined/undetermined pathogenesis).

### 2.3 Data collection

Demographic data (age and sex) and traditional risk factors were collected on admission for all patients and controls. Traditional risk factors included hypertension, diabetes, coronary arteriosclerotic cardiopathy, atrial fibrillation, stroke, smoking habits, and alcohol consumption. After clinical evaluation, patients underwent a standard assessment protocol, consisting of laboratory tests, ECG, echocardiography, color Doppler ultrasonography, and neuroimaging. Neuroimaging included Magnetic Resonance Imaging (MRI) or Computed Tomography (CT) scans of head, and non‐contrast MRA with use of an Achieva 3.0T (United Kingdom Philips) scanner. The involved extracranial and/or intracranial artery stenosis associated with the fresh cerebral infarction were further divided into three groups as based upon extent of the stenosis (<50%, 50%–69%, or ≥70% stenosis).

Classifications of stroke subtypes were based on the individual characteristics of the patient combined with the results from one or more of their diagnostic tests, including brain imaging (CT and MRI), ECG, echocardiography, color Doppler ultrasonography, and laboratory tests for a prothrombotic state. The subtype classifications were completed by two neurologists who were from the Department of Neurology of the First Affiliated Hospital of Harbin Medical University.

Blood was withdrawn prior to the initiation of therapy. According to CHANCE trials (Wang et al., (2013), the patients with minor stroke were treated with a combination therapy consisting of clopidogrel and aspirin. Clopidogrel and aspirin do not influence PTX3 levels (Korybalska et al., 2018; Omote et al., 2014). Antihypertensive drugs, insulin, and oral hypoglycemic agents were used for controlling blood hypertension and diabetes mellitus. Thrombectomy and acute stent placement in the stenotic vessels or thrombolysis were not applied in patients during the acute phase of their stroke.

### 2.4 Outcomes

We used modified Rankin Scale (mRS) (Swieten et al., 1988) to determine functional outcomes at 90 days after admission. Functional outcome was defined as favorable (mRS ≤ 2) or unfavorable (mRS score > 2). A certified mRS assessor who was blind as to serum PTX3 levels participated in mRS scoring either through an in‐person visit or by phone.

### 2.5 Enzyme‐linked immunosorbent assay for PTX3

Blood samples were collected within 48 h rafter onset of stroke in patients who had fasted for 12 hr, and after centrifugation serum samples were then frozen at −80℃. Blood samples from controls were collected contemporaneously with that of the patients. Serum PTX3 levels were determined using a quantitative sandwich enzyme‐linked immunosorbent assay kit (Cat#CSB‐E12926h, Cusabio Biotech) according to the manufacturers' instructions. Minimal levels of detection for PTX3 were 0.156ng/ml. The intra‐assay coefficient of variation (CV) was <8% and the inter‐assay CV was <10%.

### 2.6 Statistical analyses

Results are expressed as percents for categorical variables, as means with standard deviation for variables showing a normal distribution or medians for variables failing to show a normal distribution. In univariate analyses, continuous variables were compared using Student's *t* tests for normally distributed variables, Mann–Whitney *U* tests for variables failing to show a normal distribution between the groups and categorical variables with use of a Chi‐Square test. In multivariate analyses, we performed logistic regression analyses with adjustment for confounding factors. Two‐sided *p* levels <.05 were required for results to be considered statistically significant. All statistical analyses were performed using the SPSS, version 19.0, package for windows (SPSS Inc.).

## 3 RESULTS

In this study, 246 consecutive patients with acute minor stroke were initially recruited and 241 completed the 90‐day follow‐up (five patients failed to be contacted). Background characteristics of control subjects (*N* = 241) and stroke patients are summarized in Table 1. No significant differences between these two groups were present with regard to hypertension, diabetes mellitus, coronary heart disease, atrial fibrillation, smoking, and alcohol consumption. Serum PTX3 levels were significantly increased (*p* < .001) in stroke patients (3.14 ± 1.23 ng/ml) as compared with those in the control group (2.44 ± 0.74 ng/ml; Table 1). Serum CRP levels were also significantly greater (*p* = .043) in stroke patients (1.53 ± 0.38 ng/ml) as compared with those in the control group (1.35 ± 0.35 μg/ml; Table 1).

**TABLE 1:** Background characteristics of controls and stroke cases

	Controls (*n* = 241)	Cases (*n* = 241)	*p* value
Age, years, mean ± *SD*	60.85 ± 10.89	61.27 ± 10.37	.991
Male, *n* (%)	182 (75.5)	180 (74.7)	1
Risk factors			
Hypertension (%)	104 (43.2)	134 (55.6)	.119
Diabetes mellitus (%)	40 (16.6)	55 (22.8)	.389
Atrial fibrillation (%)	4 (1.7)	9 (3.7)	.721
Coronary heart disease (%)	16 (6.6)	34 (14.1%)	.170
Smoking (%)	80 (33.2)	117 (48.5)	.059
Alcohol consumption (%)	56 (23.2)	74 (30.7)	.335
Laboratory measurements			
PTX3 level (ng/ml)	2.44 ± 0.74	3.14 ± 1.23	<.001^†^
CRP (μg/ml)	1.35 ± 0.35	1.53 ± 0.38	.043*

Note

*p* values were obtained with use of the Student's *t* test for normally distributed variables and the Chi‐Square test for comparisons of proportions.

Abbreviations: CRP, C‐reactive protein; PTX3, pentraxin 3.

* *p* < .05.

^†^ *p* < .001.

On the basis of the modified TOAST classification employed, SAO was the most frequent subtype (49.3%), followed by LAA (32.0%) in our stroke patients (Table 2). Serum PTX3 values within all four of these subtypes were significantly greater than those in corresponding controls (data not shown). No statistically significant differences (*p* > .05) in serum PTX3 were obtained among the four subtypes (Table 2).

**TABLE 2:** Comparisons of PTX3 levels within the Trial of Org 10172 in Acute Stroke Treatment (TOAST) subgroups

TOAST classification	PTX3 level (ng/ml)	Cases (%)
Small‐artery occlusion (SAO)	3.125 ± 1.158	119 (49.3)
Large artery atherosclerosis (LAA)	3.159 ± 1.157	77 (32)
Cardioembolic infarction (CE)	3.008 ± 1.700	8 (3.3)
Other type of brain infarction (OT)	3.153 ± 1.499	37 (15.4)
Sum		241 (100)

Note

*p* values were obtained with use of Kruskal–Wallis and chi‐squared tests. There were no statistically significant differences (*p* > .05) among the four TOAST subgroups (LAA, SAO, CE, and OT).

Abbreviation: PTX3, pentraxin 3.

The 241 stroke patients were classified into a favorable and unfavorable outcome group. An unfavorable outcome was observed in 31 patients (12.9%) and a favorable outcome in 210 patients (87.1%). As based on results from univariate analysis, serum PTX3 levels, diabetes mellitus, discharge NIHSS score and a intracranial artery stenosis of ≥50% were identified as predictors for an unfavorable outcome (Table 3). Results from multivariate regression analysis indicated that diabetes mellitus and discharge NIHSS score were independently associated with an unfavorable outcome of acute minor stroke. After multivariate adjustment, the association between diabetes mellitus and an unfavorable outcome was statistically significant with an OR of 2.903 (95% CI, 1.262–6.676; *p* = .012), and discharge NIHSS score was also found to be an independent predictive factor with an OR of 1.862 (95% CI, 1.145–3.028; *p* = .012) (Table 4). PTX3 was not an independent risk factor for an unfavorable outcome of acute minor stroke.

**TABLE 3:** Univariate analysis for 90‐day outcomes in patients with acute minor stroke

Characteristic	Favorable (*N* = 210)	Unfavorable (*N* = 31)	*p* value
Demographic feature			
Male, *n* (%)	159 (75.7)	21 (67.7)	.464
Age‐year, (mean ± *SD*)	60.83 ± 10.41	64.23 ± 9.73	.093
Medical history and examination			
Current smoker, *n* (%)	103 (49.0)	14 (45.2)	.832
Alcohol abuse, *n* (%)	64 (30.5)	10 (32.3)	1
History of stroke, *n* (%)	76 (36.2)	17 (54.8)	.073
Hypertension, *n* (%)	113 (53.8)	21 (67.7)	.206
Diabetes mellitus, *n* (%)	42 (20.0)	14 (45.2)	.004*
History of atrial fibrillation, *n* (%)	9 (4.3)	0 (0.0)	.505
History of CHD, *n* (%)	28 (13.3)	6 (19.4)	.533
Admission NIHSS score, mean	1.42 ± 1.04	1.68 ± 0.91	.164
Discharge NIHSS score, mean	0.69 ± 0.75	1.26 ± 0.89	<.001^†^
Laboratory measurements			
Serum PTX3 level, ng/ml	3.09 ± 1.25	3.42 ± 1.00	.042*
Serum CRP level, μg/ml	1.53 ± 0.39	1.63 ± 0.30	.447
WBC count (×10^9^/L), mean	7.31 ± 1.94	7.61 ± 2.34	.679
Neutrophil percent, mean	66.49 ± 12.36	67.29 ± 10.53	.482
Anemia, *n* (%)	9 (4.3)	1 (3.2)	1
PLT count (*10^12^/L), mean	221.90 ± 55.33	211.87 ± 57.75	.453
PT‐INR, mean	1.03 ± 0.07	1.05 ± 0.08	.228
Serum fibrinogen, g/L, mean	2.59 ± 0.77	2.53 ± 0.76	.97
Serum creatinine, μmol/L, mean	67.69 ± 18.33	64.57 ± 18.49	.271
Serum uric acid, μmol/L, mean	331.75 ± 90.08	341.67 ± 91.74	.603
Fasting blood glucose, mmol/L, mean	5.69 ± 1.95	6.31 ± 2.36	.113
Total cholesterol, mmol/L, mean	4.77 ± 1.15	5.10 ± 1.14	.214
Serum LDL cholesterol, mmol/L, mean	3.01 ± 0.78	3.21 ± 0.53	.056
Serum triglyceride, mmol/L, Mean	1.88 ± 1.32	2.45 ± 1.92	.114
Extracranial artery stenosis ≥50%, *n* (%)	30 (14.3)	8 (25.8)	.168
Intracranial artery stenosis ≥50%, *n* (%)	57 (27.1)	16 (51.6)	.011*
Serum HDL cholesterol, mmol/L, mean	1.70 ± 7.72	1.16 ± 0.23	.69
Serum homocysteine, μmol/L, mean	16.03 ± 10.59	13.56 ± 4.28	.603

Note

*p* values were obtained with use of Mann–Whitney U tests, Student's *t* tests for continuous variables and Chi‐Square tests for categorical variables.

Abbreviations: CHD, coronary heart disease; LDL, low‐density lipoproteins; NIHSS, National Institutes of Health Stroke Scale; PT‐INR, prothrombin time and International Normalized Ratio; TC, total cholesterol; WBC, leukocytes.

* *p* < .05.

^†^ *p* < .001.

**TABLE 4:** Multivariate regression analysis of 90‐day prognosis in patients with acute minor stroke

Prediction	*p* value	*OR* value	95% CI
Serum PTX3 level, ng/ml	.165	1.243	0.914–1.689
Discharge NIHSS score	.012*	1.862	1.145–3.028
Diabetes	.012*	2.903	1.262–6.676
Intracranial artery stenosis ≥ 50%	.150	1.867	0.798–4.371

Note

*p* for multivariate models. Data are presented as ORs (95% confidence interval).

Abbreviations: NIHSS, National Institutes of Health Stroke Scale; PTX3, pentraxin 3.

* Indicates *p* < .05.

Within the TOAST subgroups, the relationship between PTX3 levels and unfavorable outcomes was determined only for the LAA and SAO subgroups as sample sizes in the CE (*N* = 3) and OT (*N* = 1) subgroups were too small for such calculations. Within the LAA subgroup (*N* = 77), there were 17 patients classified as experiencing an unfavorable outcome and 60 with a favorable outcome. As based on univariate analysis, serum PTX3 levels, diabetes mellitus, discharge NIHSS score, and serum LDL cholesterol (LDL‐C) were predictors for an unfavorable outcome in this LAA subgroup (Table 5). Results from multivariate logistic regression analysis revealed that serum PTX3 and LDL‐C levels could predict an unfavorable outcome at day 90 in these LAA patients with ORs of 1.169 (95% confidence intervals: 1.037–2.886) and 3.075 (95% confidence intervals: 1.189–9.089), respectively (Table 5). There were no statistically significant differences in serum PTX3 levels between a favorable versus unfavorable outcome in the SAO subgroup (data not shown).

**TABLE 5:** Univariate analysis and multiple regression analyses of predictors in the LAA subgroup

	Univariate			Multiple		
	OR (CI 95%)	*Β*	*p*	OR (CI 95%)	*Β*	*p*
Serum PTX3 level	1.189 (1.151–2.988)	0.598	.010^†^	1.169 (1.037–2.886)	0.526	.040*
Diabetes mellitus	3.214 (1.026–10.142)	1.167	.043*			
Serum LDL Cholesterol	2.311 (1.033–5.598)	0.838	.049*	3.075 (1.189–9.089)	1.123	.028*
Discharge NIHSS score	1.874 (1.043–3.526)	0.628	.040*			

Note

A univariate and backward stepwise multiple Cox regression analysis were performed to identify independent predictors within the LAA subgroup.

Abbreviations: LAA, large artery atherosclerosis; LDL, low‐density lipoproteins; NIHSS, National Institutes of Health Stroke Scale; PTX3, pentraxin 3.

* *p* < .05.

^†^ *p* = .010.

**FIGURE 1:**
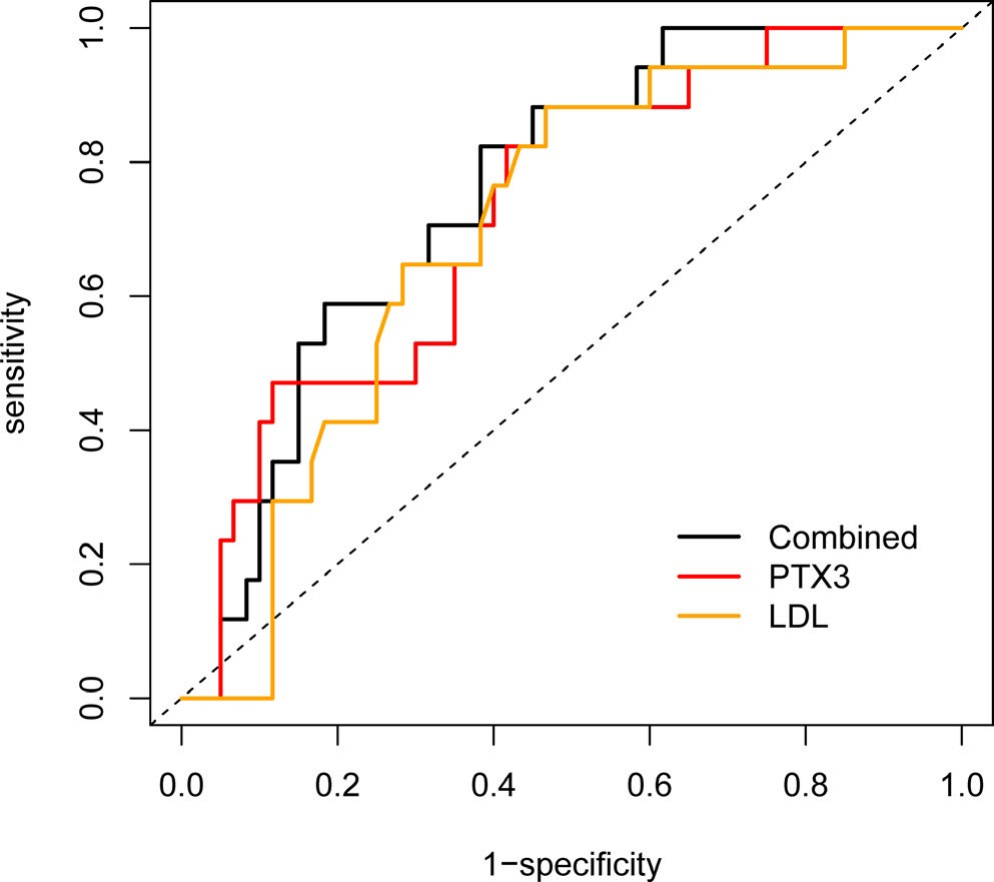
Predictive value of serum PTX3 levels in the LAA subtype of acute minor stroke. AUC, area under the curve; LAA, large artery atherosclerosis; LDL, low‐density lipoproteins; PTX3, pentraxin 3. ROC curves were generated based on levels of serum PTX3 and LDL cholesterol as predictors of an unfavorable outcome in patients with acute minor stroke. The AUCs in PTX3 were 0.726 (95% CI, 0.598–0.855), 0.701 (95% CI, 0.574–0.829) in LDL cholesterol and 0.756 (95% CI, 0.642–0.870) in combined PTX3 and LDL

Receiver operating characteristic (ROC) curve analyses were then performed for the variables showing a statistically significant association with an unfavorable outcome in the LAA subgroup. The AUCs at the 95% CI for these ROC analyses were 0.726 (0.598–0.855) for PTX3 and 0.701 (0.574–0.829) for LDL‐C. No statistically significant differences were obtained between PTX3 and LDL‐C in predicting an unfavorable outcome in the LAA subgroup (*p* = .787; Figure 1).

## 4 DISCUSSION

To our knowledge, the results of this study are the first to show a significant association between serum levels of PTX3, a marker of inflammation, and an unfavorable outcome in the LAA subtype of acute minor stroke patients. Although serum PTX3 levels were equally increased in all stroke subtypes, the clinical significance of such increases may be quite different among these subgroups. For example, we found that a significant association between levels of PTX3 and functional outcome was present only in LAA patients, with increased PTX3 concentrations being associated with an increased risk for an unfavorable outcome in this subgroup. As based on the TOAST system, the LAA subgroup is defined as comprising patients with a ≥50% intracranial/extracranial artery stenosis or occlusion, cortical or cerebellar lesions and brain stem or subcortical hemispheric infarcts >1.5 cm in diameter (Adams et al., 1993). As PTX3 is produced by cells involved with atherosclerotic lesions responding to the toll‐like receptor agonists, TNF‐a, IL‐1b, and other inflammatory mediators, PTX3 reflects local inflammatory reactions that can be detected in human carotid atherosclerotic lesions (Soeki & Sata, 2016). Therefore, given that PTX3 is considered an important marker of vascular pathology, it seems probable that serum PTX3 levels can serve as a predictor of an unfavorable outcome at day 90 in LAA patients.

In this study, not only were levels of PTX3 significantly elevated in these acute minor stroke patients, but CRP as well. PTX3 is one of the pattern‐recognition receptors implicated in the initial steps of complement cascade activation prior to CRP (Garlanda et al., 2005). Although CRP is a common acute‐phase reactive protein that can be used to assess inflammation in clinical settings, as a type of marker for systemic inflammation, it shows poor specificity. Accordingly, conflicting evidence exists as to whether increased CRP levels may be associated with stroke severity on admission and functional outcome at discharge (Irimie et al., 2018). In this study, we found that only PTX3, but not CRP levels, were significantly correlated with an unfavorable outcome in the LAA subgroup of acute minor stroke. PTX3 can reflect different aspects of atherosclerosis‐related inflammation than that revealed by CRP and may thus provide additional insight into atherosclerosis development and progression (Jenny et al., 2014). As a result, when considering its interactions with atherosclerosis conditions such as that occurring in acute ischemic stroke, PTX3 appears to be of superior prognostic value as compared to CRP in the LAA subgroup.

In our study, we found no statistically significant differences in serum PTX3 levels between a favorable versus unfavorable outcome in the SAO subgroup. SAO, defined as comprising patients showing a clinical syndrome of acute stroke and an infarct size of ≤15 mm in diameter, may result from lipohyalinosis or a distinct and specific small‐vessel arteriopathy without atheroma occluding a single perforating artery at the base of the brain as well as in larger arteries (Adams et al., 1993). To our knowledge, there currently exist no reports indicating an association between SAO and serum PTX3 levels. Clinically, overt vascular inflammation involving small or large vessels has been associated with elevated PTX3 levels in acutely inflamed patients with various systemic autoimmune and inflammatory conditions. Although PTX3 represents a novel acute‐phase reactant produced at sites of active vasculitis and correlates with disease activity (Ramirez et al., 2019), the vasopathology of SAO is completely different from that of vasculitis.

We also found that serum PTX3 and LDL‐C levels proved to be independent predictors of an unfavorable outcome in the LAA subtype, with no statistically significant differences being obtained between PTX3 with LDL‐C levels as determined by ROC analysis. When PTX3 and LDL‐C levels were combined for this analysis, an increased AUC in the ROC index was obtained as compared with that for either PTX3 or LDL‐C alone, indicating that this combination proved a better predictor for an unfavorable outcome in the LAA subtype. PTX3 and LDL‐C have also been shown to correlate with the prevalence and severity of carotid artery stenosis, which suggests a mechanism linking LDL to inflammation mediators (Yi et al., 2020). In patients with hypercholesterolemia, PTX3 correlated with the severity of vascular disease and statin therapy decreased plasma PTX3 levels, which suggests an involvement of PTX3 in vascular inflammation triggered by LDL‐C (Ohbayashi et al., 2009). However, PTX3 may also exert an atheroprotective role as revealed in a sample of the general population from a community‐based cohort. PTX3 levels have been reported to be positively associated with HDL‐C levels and negatively associated with reduced LDL/HDL‐C ratios (Lee et al., 2019). HDL‐induced PTX3 expression in the endothelium, which is dependent on the PI3K/Akt pathway, may explain the vascular‐protective effect of PTX3 (Norata et al., 2008). We believe that PTX3 may play different roles in atherosclerosis between stroke patients and the general population, which can result from complex effects of PTX3 upon the vasculature due to the involvement of multiple pathways.

The immune response to acute cerebral ischemia is a major factor in stroke pathobiology and outcome. While the immune response initially starts locally in occluded and hypoperfused vessels and the ischemic brain parenchyma, the subsequent in situ generation of inflammatory mediators then propagates throughout the entire organism (Anrather & Iadecola, 2016). Although not constitutively expressed in the central nervous system, PTX3 transcription has been shown to be amplified in acute ischemic stroke (Katakami et al., 2013). The exact mechanisms accounting for the association of PTX3 with an unfavorable outcome in the LAA subtype of acute minor stroke remain unclear. The findings that PTX3 promotes long‐term recovery of cerebral blood flow, angiogenesis, and neuronal viability after cerebral ischemia suggest the potential for PTX3 as a promising therapeutic target of clinical relevance (Rajkovic et al., 2018). In contrast to that of experimental evidence from animal studies, PTX3 has been shown to positively correlated with stroke severity and provides an indication for poor prognosis and prediction of mortality after ischemic stroke. PTX3 may also be an active mediator of endothelial dysfunction and atherosclerotic plaque vulnerability (Ryu et al., 2012; Sezer et al., 2014). In one study directed at identifying a role for PTX3 in thrombolytic therapy in acute ischemic stroke, no association between PTX3 and neurological improvement and long‐term prognosis were observed (Zhang et al., 2018). Therefore, substantial evidence exists suggesting a dual role for PTX3 as a modulator or amplifier of the innate immune response. The final effects of PTX3 activation might be determined by a fine tuning resulting from supplementary cues of time, space, and environmental signals (Casula et al., 2017).

The present study has some limitations. Our study involved a relatively small sample size of patients that were predominantly male and studied within a single‐center. As the study was conducted at a single‐center in China, the acute minor stroke patients recruited might possess a number of differences in characteristics compared with those in Western studies. Information on infarct volume was not included in this study, nor was the time from symptom onset to MRI available. As DWI lesion volumes change with time, not adjusting for time from ictus to MRI may affect the lesion volumes calculated. Based upon these limitations, a large, multicenter study will be required to confirm our results. Despite these limitations, we believe our study provides new and important data revealing the first evidence for the prognostic significance of PTX3 in acute minor stroke.

In conclusion, in this study we show that increased serum PTX‐3 levels might be used as an independent predictor of an unfavorable outcome of acute minor stroke within the LAA subgroup. Accordingly, determinations of PTX3 levels may provide important prognostic information in these patients. Overall, the prognostic value of PTX3 within the LAA subgroup proved superior to that of other members of the pentraxin family like CRP. In order to further elucidate a role for PTX3 in risk prediction, additional studies will continue to assess the significance of this biomarker.

## CONFLICTS OF INTEREST

The authors declare no conflict of interest.

## AUTHOR CONTRIBUTIONS

Yan Zhang involved in conceptualization, methodology, and editing. Haijie Hu involved in data curation and writing the original draft preparation. Chong Liu involved in data curation, investigation, and software. Juan Wu involved in validation and supervision. Shanshan Zhou involved in writing, reviewing, and visualization. Tingting Zhao involved in software and visualization.

### Peer Review

The peer review history for this article is available at https://publons.com/publon/10.1002/brb3.1956.
